# Systematic and meta-based evaluation on job satisfaction of village doctors: An urgent need for solution issue

**DOI:** 10.3389/fmed.2022.856379

**Published:** 2022-08-18

**Authors:** Yuquan Chen, Yanwei You, Yue Wang, Yudong Wang, Tao Dai

**Affiliations:** ^1^Institute of Medical Information and Medical Library, Chinese Academy of Medical Sciences, Beijing, China; ^2^Peking Union Medical College, Beijing, China; ^3^School of Social Sciences, Tsinghua University, Beijing, China

**Keywords:** job satisfaction, village doctors, evidence-based decision making, health policy, meta-analysis

## Abstract

**Background:**

Village doctors are the health “gatekeepers” of rural residents in most developing countries. They undertake a series of strenuous but pivotal missions, including prevention, diagnosis, and treatment of complicated diseases, sanitation services and management, and preventive healthcare and education tasks. Hence, it is of great importance to evaluate the village doctors’ job satisfaction status, which is one of the most important indicators that can reflect the current working state, to provide guidelines for the healthcare policies.

**Methods:**

Literature search was conducted in 7 authoritative databases, including PubMed, EMBASE, Web of Science, and China National Knowledge Infrastructure (CNKI). Experts in the field of social medicine were consulted to achieve supplement and obtain relevant literature. China was selected as a representative of the village doctor system for the in-depth analysis. Building on the previous literature, we modified and proposed a novel strategy that can transform and integrate the outcome indicators to conduct a meta-based and quantitative assessment on job satisfaction.

**Results:**

A total of 37 publications and 23,595 village doctors were included in this research. The meta-analysis showed that the overall job satisfaction score of village doctors was 3.1858 (total score: 5.00), 95% *CI*: 2.9675–3.404, which represented the level of “neither satisfied nor dissatisfied.” However, in the subsequent adjustment of publication bias, this score reduced to 2.7579, 95% *CI*: 2.5254–2.9904, which indicated a direct “dissatisfied” level. To discover the underlying causes, a holistic analysis of each dimension and influencing factors of job satisfaction was conducted, and the results demonstrated that “Financial Rewards” (2.49) was the most important factor causing dissatisfaction among village doctors, followed by “Job Security (2.52)” and “Work Stress (3.05).” Several important themes were also identified and assessed to explore the factors related to this topic.

**Conclusion:**

This study indicated that there is an urgent need to improve the working status of health workers in rural and remote areas, especially in the middle- and low-income countries. Health policy makers should not only improve the current remuneration and subsidies of village doctors but also guide the professional development and give them more job security to enhance the work stability of this group. More specifically, in the context of the COVID-19 pandemic, further surveys on job satisfaction of village doctors should be carried out to take targeted measures.

**Systematic review registration:**

[https://www.crd.york.ac.uk/PROSPERO/], identifier [CRD42021289139].

## Introduction

Village doctors, who are affectionately known as “gatekeepers” of the rural health service systems, refers to the personnel who have obtained the qualification certificate of village doctors and work in village clinics (1). They are the guardians of people who are living in rural and remote areas (2, 3) and provide basic public health services, mainly including the establishment of rural health archives, education of health knowledge, prevention and control of infectious diseases, healthcare for the elderly, and management of chronic diseases. As the most basic and extensive medical service provider in rural areas, village doctors play an irreplaceable role in ensuring and improving the health level of rural residents.

In most of the developing countries, the medical technology level and service quality of village clinics have been falling behind during the past years due to the lack of official funding. More seriously, the reform of the health system schedule also excluded village doctors from the government project, resulting in not only the low satisfaction of rural residents with medical services but also the poor satisfaction of village doctors themselves (4). Among various developing countries, village doctors in China have a long history. Since the 1950s, there has been a tradition of barefoot doctors (5, 6). One previous research in The Lancet supported that “The barefoot doctor system was considered as a successful example of healthcare provision in developing regions with in-adequate resources by World Health Organization (WHO)” (7). Since entering the twenty-first century, public health has received more attention and it is of great significance to review and evaluate this issue under new situations and circumstances.

Job satisfaction refers to the extent to which people prefer (satisfaction) or refuse (dissatisfaction) their jobs, and can reflect the attitude or emotional response to the workplace (8, 9). It is one of the most important predictors of medical staff burnout, which is a kind of psychological syndrome (10) that refers to a series of psychological and physiological reactions caused by the pressure of interpersonal relationships and work itself. Turnover intention refers to the idea that an individual has to resign from their current job and look for another job (11). In the classical turnover theory, turnover intention is usually regarded as an important cognitive process before turnover behavior. A survey of 1,148 primary care providers (P) in a rural district found that there was a significant direct effect of job satisfaction on burnout and turnover intention, a significant direct effect of burnout on turnover intention, and a significant indirect effect of job satisfaction on turnover intention through burnout as a mediator (12). Another study on job burnout, satisfaction, and turnover intention of primary healthcare staff also confirmed this viewpoint (13).

At present, there are only a few studies on the job satisfaction of village doctors in specific areas or a small range. On one hand, scholars are more interested in the evaluation of the current situation or influencing factors of job satisfaction (9, 14–18), and there are more relevant studies on health workers in urban public hospitals rather than village doctors. On the other hand, different studies may use different methods to evaluate job satisfaction: some scholars directly put forward the concept of overall job satisfaction (19–24), while others (25–28) reported their job satisfaction in multidimensional items (including income satisfaction, work environment satisfaction, and leadership satisfaction). In addition, different surveys reported diverse evaluation scales with several parts in the form of satisfaction distribution (29–33) and others in the form of scores (19–22, 24, 34). Therefore, the realistic dilemma for this research topic is that the evidence on the job satisfaction survey of villager doctors is fragmented and it is not a piece of cake to provide a high-quality reference for the formulation of national policies from an overall and systematic perspective.

Consequently, based on the above situation, we aimed to systematically evaluate the meta-based evidence of job satisfaction in order to provide an important reference basis for stabilizing the structure of rural doctors and improving the development of human resources in the primary medical system as well as the quality of health services. Simultaneously, we also targeted to use the cases in China to present a unique perspective for the systematic review of healthcare workers’ job satisfaction in other developing countries or regions worldwide.

## Materials and methods

This systematic review was conducted in accordance with the Preferred Reporting Items for Systematic Reviews and Meta-Analysis Protocols guidelines (35) and is registered with the International Prospective Register of Systematic Reviews (PROSPERO, registration number: CRD42021289139).

### Search strategy

A total of 7 databases were searched by computer, including PubMed, Embase, Web of Science, China National Knowledge Infrastructure (CNKI), WanFang, China Science and Technology Journal Database (VIP), and Chinese BioMedical Literature Database (CBM). In addition, experts in the field of social medicine were consulted to achieve supplement and obtain relevant literature. Pre-retrieval results showed that there were few publications before 2011. Consequently, the retrieval time limit was set from 01 January 2011 to 01 December 2021 so as to better reflect the latest situation. The search strategy was based on a combination of “rural doctor,” “rural physician,” “village doctor,” “village physician,” “work satisfaction,” “job satisfaction,” “career satisfaction,” and so on. The specific literature retrieval strategies of each database can be found in Supplementary Appendix A.

### Study eligibility

Eligible studies were published literature that reported the prevalence or questionnaire score and related determinants of job satisfaction among village doctors in China. The eligibility criteria included the following: (1) Types of studies: original cross-sectional studies (those presenting non-original data, such as reviews, editorials, opinion papers, or letters to the editor, were excluded); (2) Types of participants: Chinese village doctors; (3) The outcome of job satisfaction measures: the status of job satisfaction (including the distribution of the number of people or score) and its related dimensions were reported. Satisfaction dimensions were diverse in different studies, so they should be grouped by dimensions by conceptual affinity. Closely related dimensions were categorized as one theme (36). All dimensions were formed by reciprocal (supporting or complementing each other) or opposite arguments. (4) The studies that were repeatedly published or whose data information is incomplete and the relevant data cannot be obtained or missing should be excluded.

### Data extraction

First, title information for relevant literature was retrieved through the search strategy, and the Endnote *X9* software was used for literature management. After the duplication process of publications included in, two reviewers read the title and abstract for preliminary screening according to the inclusion and exclusion criteria, and then further read the full text to judge the qualification. Disagreements about the inclusion criterion were resolved by a third reviewer. For the qualified literature finally selected, two parallel groups independently extracted the research data and made records, including the first author, survey time, survey area, sampling method, number of satisfied participants, and score of satisfied participants.

### Quality assessment

In the study, two reviewers independently evaluated the risk of bias and cross checked the results. When the two reviewers show different opinions, the third reviewer shall decide by discussion. The quality of cross-sectional studies was evaluated by using 11 items of the observational study quality evaluation standard recommended by American healthcare quality and research institutions (37). The total score was 11 points, and all the included studies were grouped according to their scores, which were categorized as good (8–11), moderate (4–7), and poor (0–3). The risk of bias (ROB) of the original study was determined according to the quality of the results.

### Data synthesis and statistical analysis

This study applied a meta-based strategy to systematically review the research status of job satisfaction. Unlike other quantitative analysis from documentary ways such as bibliometric (38, 39), meta-analysis can not only provide an evidenced-based insight but also assess the consistency of multiple research results on the same subject, which may help to have a more accurate and objective evaluation of effect indicators and explain the heterogeneity among different research results.

The primary outcome in this review was the difference and status in the score about kinds of dimensions of job satisfaction among groups. The proportion was estimated as the total number of positive cases (i.e., the number of village doctors satisfied with their current job) divided by the total number of participants. The evaluation of job satisfaction is relatively complicated, which is due to the high heterogeneity of the original questionnaire used in each study. To solve this problem, we transformed different calculations of overall and kinds of dimensions of job satisfaction into a common rubric of five-point scores. Original five ratings for satisfaction were as follows: (1) “very dissatisfied” (VD), (2) “dissatisfied” (DS), (3) “not sure” (NS), (4) “satisfied” (ST), and (5) “very satisfied” (VS). Each of satisfaction levels was converted into five-point scores (VD: 1.00, DS: 2.00, NS: 3.00, ST: 4.00, and VS: 5.00). Furthermore, we defined intervals for satisfaction scores as follows: scores from 0 to 1.99 were defined as VD, 2.00–2.99 as DS, 3.00–3.49 as NS, 3.50–3.99 as ST, and 4.00–5.00 as VS (5). Referring to previous literature, we mainly applied three formulas in the process of conversion (40): (1) Satisfaction = VD% × 1.00 + DS% × 2.00 + NS% × 3.00 + ST% × 4.00 + VS% × 5.00. This formula can convert the number distribution of satisfaction into the corresponding score, which applies to the situation where only the number distribution of overall satisfaction or its dimensions is reported, and the original scale should be a 5-point Likert scale. (2) Satisfaction = reported score/maximum total score × 5.00. This formula can convert the satisfaction dimension of different total scores into a 5-point system in equal proportion. For example, with respect to the questionnaire with a total score of 100, if the reported satisfaction score is 50, the converted score is 50/100 × 5.00 = 2.50. (3) Satisfaction = DS% × 1.50 + NS% × 3.00 + ST% × 4.50. The scope of application of this formula is the same as the first formula, whereas the original scale should be a 3-point Likert scale.

The meta-package in the *R* software (version 4.0.3, Auckland University, United States) was used for data analysis, and a single-arm strategy was applied to assess the results achieved. For the calculation of proportion, first, the normality test was conducted. If the data did not conform to normality, it would be transformed by logarithm, logit, or double anti-sinusoidal transformation. For the evaluation of scores, we used the inverse variance weighting method to combine. Referring to the website StatsToDo (41), when multiple dimensions of a certain theme of job satisfaction are reported simultaneously in the same study, the mean and standard deviation of all dimensions will be combined into a set of values and further combined with other literature using the meta-analysis method.

The Cochrane *Q*-test and *I*^2^-value were used to test whether there was heterogeneity among all studies (42). According to the Meta-Analysis of Observational Studies in Epidemiology guidelines (43), if *P* > 0.10 and *I*^2^ ≤ 50%, there was no statistical heterogeneity among the research results, and the fixed effect model was applied to analyze the results; If *P* ≤ 0.1 and *I*^2^ > 50%, the random effect model was used for the meta-analysis. Publication bias was evaluated using *Egger*’s test combined with a funnel plot. To solve potential publication bias, the trim-and-fill method was conducted to adjust for prospective plot asymmetry. Furthermore, sensitivity analysis was performed by grouping or excluding low-quality studies. If it was infeasible to make a quantitative synthesis and conduct a meta-analysis, a narrative approach and descriptive statistics were applied.

## Results

### Study and sample characteristics

**FIGURE 1:**
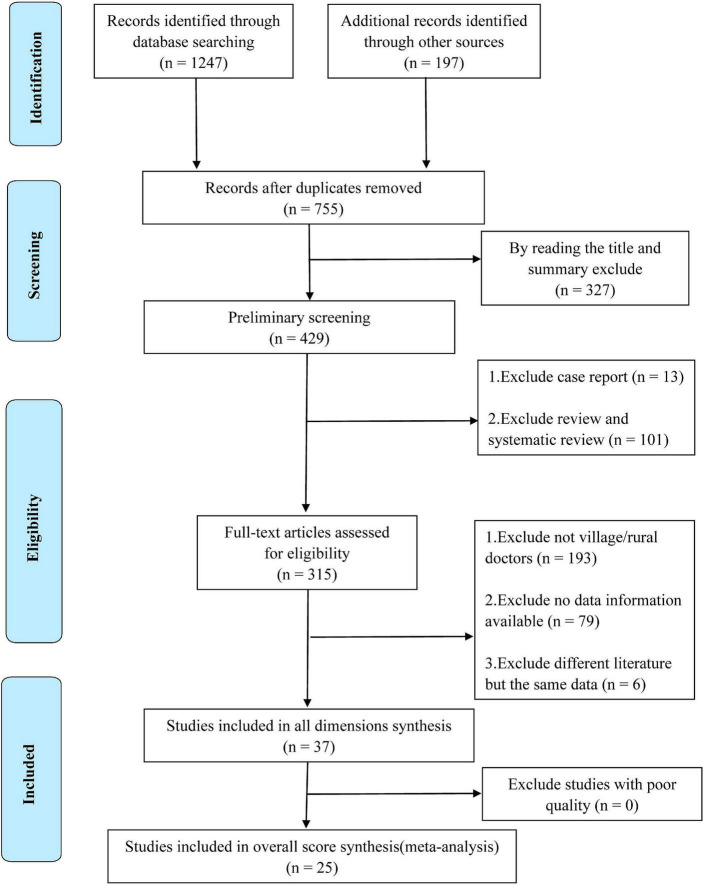
PRISMA flowchart of included studies.

A total of 1,444 studies were obtained from databases. Initially, 689 duplicates were eliminated by using the Endnote *X9* software, and then 327 irrelevant studies was eliminated by reading titles and abstracts, leaving 429 potentially qualified studies. The type of review studies, documents with inconsistent research objects, and incomplete data information were excluded by reading the full text. Finally, 37 studies (2, 3, 5, 8, 19–34, 44–60) were included for all dimensions of job satisfaction analysis. Among them, 25 studies were used to evaluate the overall score of job satisfaction (refer to Figure 1 for the detailed process).

As presented in Table 1, a total of 23,595 village doctors were included. To ensure the accuracy of the inclusion results and to include the research objects as comprehensively as possible in the research of Leiyu Shi, Ping He, and Rui Zhang (19, 22, 23, 59), we only selected the data of rural doctors reported therein for inclusion and analysis (including the number of research objects, corresponding overall job satisfaction scores, and scores of various dimensions). Except for the studies that did not report the survey time (5, 23, 26, 30, 31, 46, 47, 49, 52), the time range of the satisfaction survey basically covered the past decade. In terms of spatial scope, in addition to one study that did not report the specific geographical location (31), it included 28 provincial administrative regions in China, of which 22 studies (2, 3, 5, 8, 19–24, 27, 29, 31, 32, 34, 44, 47, 49, 52, 53, 55, 57–60) reported the satisfaction data of village doctors in eastern China, 7 studies reported the data of western China (2, 20, 26, 27, 32, 34, 53), and 3 studies reported the data of central China (2, 5, 49). Additionally, 4 studies directly used the data obtained from surveys conducted nationwide or in the central, eastern, and western regions (19, 33, 45, 54). After excluding 5 studies that did not report specific sampling methods (22, 23, 31, 49, 57), only 3 studies were conducted based on convenience sampling (8, 59, 60), 18 studies clearly reported that they were conducted based on the principle of random sampling (2, 3, 19, 21, 23, 24, 27, 28, 31, 32, 34, 45, 50–52, 55, 57), and another study was surveyed on the basis of census (54). Of all the studies, only one reported job satisfaction after the COVID-19 pandemic (53). Supplementary Appendix B shows the job satisfaction evaluation results and conversion methods of each original study.

**TABLE 1:** Characteristic of 37 included studies of the status of Chinese village doctors’ job satisfaction.

Study ID	References	Publication year	Survey area	Investigation period	Sampling method
1	Zhang et al. (3)	2021	Shandong Province	2019.05∼2019.06	Stratified cluster random sampling
2	Zhang and Fang (5)	2016	Jiangxi Province	*NA*	Multi stage stratified cluster sampling
3	Li et al. (29)	2015	Liaoning Province	2013.04∼2013.07	Randomized cluster sampling
4	Zhang et al. (2)	2019	Gansu Province and Sichuan Province	2012∼2013	Multi stage stratified sampling method
5	Gu et al. (44)	2019	Shandong Province	2016.06∼2016.08	Stratified random sampling
6	Li et al. (45)	2017	Shandong, Guangxi and Shaanxi Provinces	2014.04	Multi stage random sampling
7	Shi et al. (19)	2014	Five provinces representing Eastern, Central, and Western China	2011	Multistage stratified purposive sampling
8	Miao et al. (20)	2017	Ten administrative areas in western China	2009∼2011	Multistage stratified random sampling
9	Chen et al. (21)	2021	Shandong Province	2012,2015,2018	Multistage sampling method
10	He et al. (22)	2014	Anhui Province	2012.04	*NA*
11	Fu et al. (30)	2012	Anhui Province	*NA*	Stratified sampling
12	Li et al. (46)	2013	Beijing	*NA*	Stratified sampling
13	Zhang and Zhu (47)	2014	Jiangsu Province	*NA*	Stratified random sampling
14	Zhang et al. (23)	2014	Shaanxi Province	*NA*	*NA*
15	Zhang et al. (48)	2019	Hebei Province	2017.10∼2017.12	Stratified sampling
16	Zhang (49)	2014	Hunan Province	*NA*	*NA*
17	Sun et al. (25)	2018	Henan Province	2015.12∼2016.06	Stratified random cluster sampling
18	Sun et al. (50)	2017	Shandong Province	2015.10∼2015.11	Stratified cluster random sampling
19	Jing et al. (51)	2020	Shandong Province	2018.05	Multi stage stratified random sampling
20	Chen et al. (24)	2016	Shandong Province	2012.08∼2012.12	Multi stage stratified sampling
21	Wang (26)	2015	Ningxia Hui Autonomous Region	*NA*	Stratified random sampling
22	Bai et al. (52)	2020	Shandong Province	*NA*	Multi stage stratified random sampling
23	Zhang and Zhu (31)	2013	57 village clinics in a poor county in a mountainous area	*NA*	*NA*
24	Zhao and Zhao (53)	2021	Shaanxi Province	2020.05∼2020.06	Stratified random sampling
25	Hu et al. (34)	2011	Guizhou Province	2010.01	Census
26	He et al. (54)	2011	8 provinces in China	2010.03	Stratified random sampling
27	Dai et al. (55)	2017	Anhui Province	2016.06∼2016.07	Random sampling
28	Qu et al. (32)	2013	Ningxia Hui Autonomous Region, Sichuan and Yunnan Province	2011.05∼2011.11	Cluster sampling
29	Peng (33)	2012	8 provinces in China	Up to 2009.12	Multi stage stratified cluster sampling
30	Ma et al. (56)	2017	Shandong Province	2015.10∼2015.11	Multi stage stratified random sampling
31	Ding and Yang (57)	2020	Jiangsu Province	2019.05	*NA*
32	Shen et al. (58)	2019	Shandong Province	2018.07∼2018.08	Multi stage stratified random sampling
33	Han et al. (27)	2014	Gansu Province	2012.07	Convenience sampling
34	Wang (59)	2020	Jiangsu Province	2019.07	Stratified sampling and convenience sampling
35	Sun (8)	2017	Anhui Province	2015.09∼2017.03	Convenience sampling
36	Zhang and Zhu (60)	2014	Jiangsu Province	2013.05	Simple random sampling
37	Lu et al. (28)	2017	Shandong Province	2016.10∼2016.11	Stratified cluster random sampling

NA, not reported.

**FIGURE 2:**
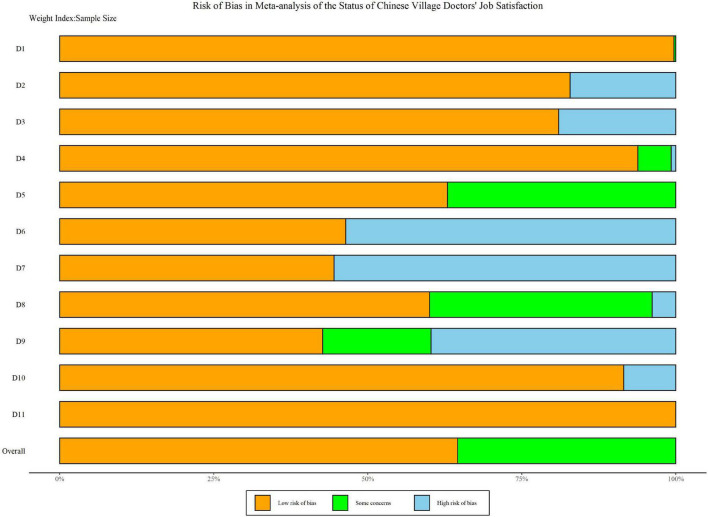
Summary plot of risk bias.

Table 2 demonstrates the quality evaluation of research methods, including 22 high-quality studies, 15 medium-quality studies, and no low-quality studies (Table 2). The average score of the quality of overall studies was 7.76, and the standard deviation was 1.32. Summary plots of the risk bias assessment are shown in Figure 2. Additionally, the concrete traffic light plot can be achieved in Supplementary Appendix C. After quality evaluation, it can be seen that there was no literature that needed to be excluded due to low quality. Therefore, the 37 studies included in the final study can be further analyzed qualitatively and quantitatively.

**TABLE 2:** Quality evaluation results of systematic review of Chinese village doctors’ job satisfaction.

Study ID	References	*D1*	*D2*	*D3*	*D4*	*D5*	*D6*	*D7*	*D8*	*D9*	*D10*	*D11*	Overall
1	Zhang et al. (3)	1	1	1	1	Unclear	1	1	Unclear	1	1	1	9
2	Zhang and Fang (5)	1	1	0	Unclear	Unclear	1	1	1	Unclear	1	1	7
3	Li et al. (29)	1	0	1	1	1	0	0	1	1	1	1	8
4	Zhang et al. (2)	1	1	1	1	1	1	0	Unclear	1	0	1	8
5	Gu et al. (44)	1	1	1	1	Unclear	0	0	Unclear	0	1	1	6
6	Li et al. (45)	1	1	1	1	1	0	1	1	0	1	1	9
7	Shi et al. (19)	1	1	1	Unclear	1	0	0	1	Unclear	0	1	6
8	Miao et al. (20)	1	1	1	1	1	0	0	1	Unclear	0	1	7
9	Chen et al. (21)	1	1	1	1	1	1	1	Unclear	Unclear	1	1	9
10	He et al. (22)	1	1	1	1	1	1	1	0	0	1	1	9
11	Fu et al. (30)	1	1	0	1	1	0	1	1	1	1	1	9
12	Li et al. (46)	1	1	0	1	Unclear	0	1	1	0	1	1	7
13	Zhang and Zhu (47)	1	0	0	1	Unclear	0	1	0	0	1	1	5
14	Zhang et al. (23)	1	1	0	1	Unclear	0	0	0	1	1	1	6
15	Zhang et al. (48)	1	0	1	1	1	1	0	1	1	1	1	9
16	Zhang (49)	1	1	0	Unclear	Unclear	0	1	1	1	1	1	7
17	Sun et al. (25)	1	1	1	1	1	1	0	1	0	1	1	9
18	Sun et al. (50)	1	1	1	1	Unclear	0	1	1	1	1	1	9
19	Jing et al. (51)	1	1	1	1	1	0	1	1	0	1	1	9
20	Chen et al. (21)	1	1	1	1	Unclear	0	1	1	1	1	1	9
21	Wang (26)	1	1	0	1	Unclear	1	0	0	0	1	1	6
22	Bai et al. (52)	1	1	0	1	1	0	0	1	0	1	1	7
23	Zhang and Zhu (60)	Unclear	0	0	Unclear	1	0	1	0	Unclear	1	1	4
24	Zhao and Zhao (53)	1	1	1	1	Unclear	0	0	1	0	1	1	7
25	Hu et al. (34)	1	1	1	1	Unclear	0	0	1	0	1	1	7
26	He et al. (54)	1	1	1	1	Unclear	1	1	1	1	1	1	10
27	Dai et al. (55)	1	0	1	1	1	1	0	Unclear	0	1	1	7
28	Qu et al. (32)	1	1	1	1	1	1	1	0	0	1	1	9
29	Peng (33)	1	1	1	1	1	0	0	Unclear	0	1	1	7
30	Ma et al. (56)	1	1	1	1	1	1	0	1	0	1	1	9
31	Ding and Yang (57)	1	1	1	1	Unclear	0	1	Unclear	1	1	1	8
32	Shen et al. (58)	1	1	1	1	1	1	0	Unclear	0	1	1	8
33	Han et al. (27)	1	1	1	1	1	0	0	1	0	1	1	8
34	Wang (59)	1	1	1	0	1	1	0	0	1	1	1	8
35	Sun (8)	1	1	1	1	Unclear	1	1	1	Unclear	1	1	9
36	Zhang and Zhu (60)	1	1	1	1	Unclear	0	1	1	0	1	1	8
37	Lu et al. (14)	1	0	1	1	1	1	0	Unclear	1	1	1	8

**D1,** define the source of information (survey and record review); **D2,** list inclusion and exclusion criteria for exposed and unexposed subjects (cases and controls) or refer to previous publications; **D3,** indicate time period used for identifying patients; **D4,** indicate whether or not subjects were consecutive if not population-based; **D5,** indicate if evaluators of subjective components of study were masked to other aspects of the status of the participants; **D6,** describe any assessments undertaken for quality assurance purposes (e.g., test/retest of primary outcome measurements); **D7,** explain any patient exclusion from analysis; **D8,** describe how confounding was assessed and/or controlled; **D9,** if applicable, explain how missing data were handled in the analysis; **D10,** summarize patient response rates and completeness of data collection; **D11,** clarify what follow-up, if any, was expected and the percentage of patients for which incomplete data or follow-up was obtained.

### Overall job satisfaction

**FIGURE 3:**
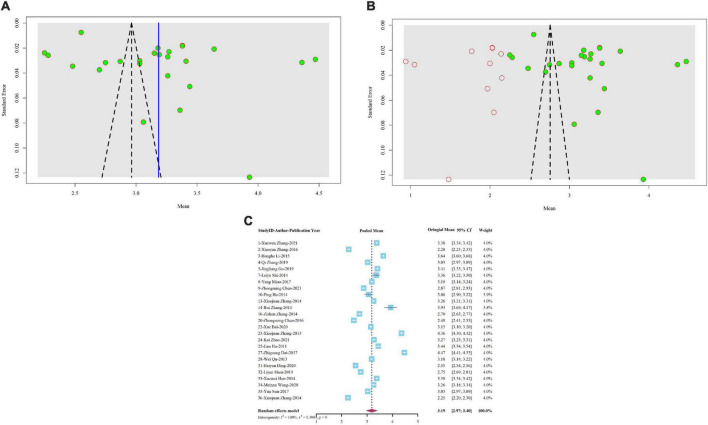
**(A)** Uncorrected funnel plot of overall job satisfaction’s meta-analysis. **(B)** Trim-and-fill corrected funnel plot of overall job satisfaction’s meta-analysis. **(C)** Forest plot of overall job satisfaction’s meta-analysis.

Of the 37 studies included, 25 proposed or reported items of overall job satisfaction. After pooling the results of 25 studies that reported overall job satisfaction by meta-analysis, the random effect size of the Chinese village doctors’ job satisfaction score was 3.1858, 95% *CI*: 2.9675–3.4041, *I*^2^ = 99.8%, and *Q* = 12,750.88 (Figure 3C), indicating that the satisfaction level was “not sure.” However, the *Egger*’s test showed that there was significant publication bias in the study of overall job satisfaction (*P* = 0.0215 < 0.05, *t* = 2.47, SE bias = 8.0591, intercept = 2.5795, and SE intercept = 0.1820). At the same time, obvious asymmetry was also observed in the funnel diagram (Figure 3A). Therefore, publication bias was corrected by the trim-and-fill method. Corrected results displayed that the total score of job satisfaction became 2.7579, 95% *CI*: 2.5254–2.9904, *I*^2^ = 99.9%, and *Q* = 28972.64. In other words, the job satisfaction level dropped to “dissatisfied” in this condition. The corrected funnel plot is shown in Figure 3B.

For 4 studies (45, 48, 51, 56) could not be transformed into formula calculation, only the proportion of people satisfied with the current job was reported in the original study (Table 1). The meta-analysis of “single arm” showed that only 43.2% of village doctors were satisfied with their current work (95% *CI*:33.1–53.2%, *I*^2^ = 97.3%, and *Q* = 110.88). After the test of publication bias, the results indicated that there was no publication bias (*P* = 0.4645 > 0.05, *t* = 0.9, SE bias = 10.7002, intercept = 0.2483, and SE intercept = 0.1717).

### Job satisfaction of each theme

All dimensions closely related to job satisfaction were summarized into 9 themes: career development, financial rewards, governance, infrastructure, interpersonal relationships, job security, respect, work value, and work stress. The different dimensions contained under each topic and the scores of which are shown in Supplementary Appendix D.

**FIGURE 4:**
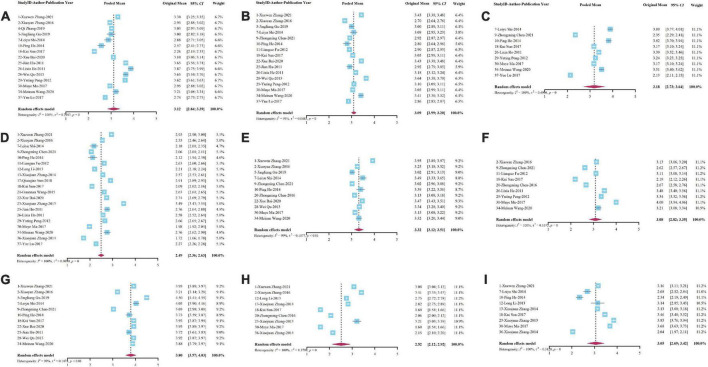
Forest plot of meta-analysis for each theme of village doctors’ job satisfaction. **(A)** Career development; **(B)** infrastructure; **(C)** respect; **(D)** financial rewards; **(E)** governance; **(F)** work value; **(G)** interpersonal relationship; **(H)** job security; and **(I)** work stress.

**FIGURE 5:**
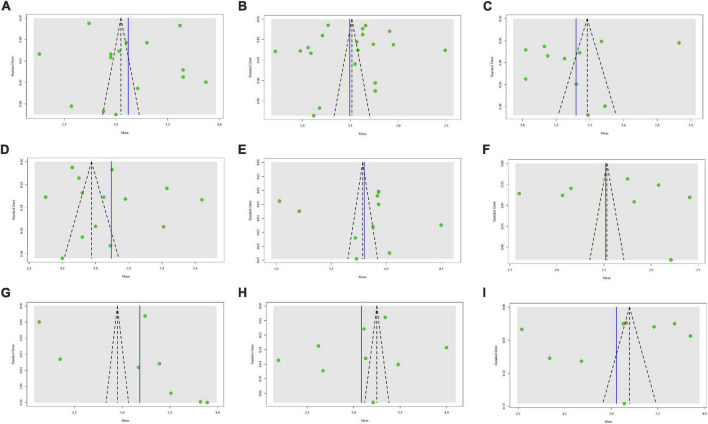
Funnel plot of each theme. **(A)** Career development; **(B)** financial rewards; **(C)** governance; **(D)** infrastructure; **(E)** interpersonal relationship; **(F)** job security; **(G)** respect; **(H)** work value; and **(I)** work stress.

**FIGURE 6:**
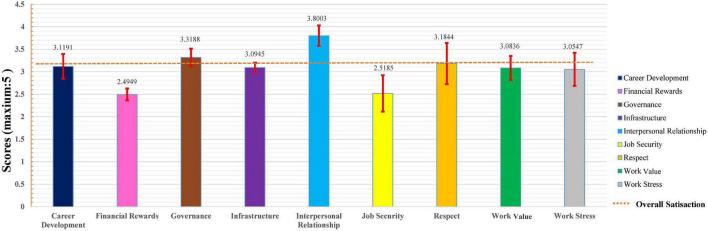
Summary results of different themes and the scores of overall job satisfaction.

The meta-analysis results of studies under each theme are displayed in Table 3, and all results were detected by publication bias. Since no publication bias occurred when the *P*-value reported by the *Egger*’s test was greater than 0.05, our results demonstrated that there was no publication bias in this case (refer to Figure 4 for the forest plot of each theme, and Figure 5 for the funnel plot of each theme). Furthermore, the summary results of different themes and the scores of overall job satisfaction are shown in Figure 6. Based on the visual comparison between the obtained result value and the picture, we could find that “financial rewards (2.49)” had the lowest satisfaction score, which was at the level of “dissatisfied,” and another theme at the same level was “job security (2.52).” Instead, the theme with the highest satisfaction score was “interpersonal relationship (3.80),” which was at the level of “satisfied,” and only this theme was at this level. Other themes were consistent with the overall job satisfaction, at the level of “not sure,” and were ranked from high to low as follows: “governance (3.32),” “respect (3.18),” “career development (3.12),” “infrastructure (3.09),” “work value (3.08),” and “work stress (3.05).”

**TABLE 3:** Meta-analysis summary of score values of different themes.

Theme	NO.*	*I*^2^ (%)	*Q*	t*	E.P*	Mean	95% *CI*
Career development	15	99.9	12021.59	0.4	0.6951	3.1191	2.8447∼3.3935
Financial rewards	20	99.7	6831.21	0.1	0.9218	2.4949	2.3629∼2.6269
Governance	11	99	1009.35	–0.95	0.3686	3.3188	3.1227∼3.5148
Infrastructure	16	99.3	2279.14	1.66	0.1192	3.0945	2.9891∼3.1999
Interpersonal relationship	11	99.3	1432.79	–0.02	0.9873	3.8003	3.5720∼4.0286
Job security	9	99.8	4356.77	–0.45	0.6675	2.5185	2.115∼2.9219
Respect	9	99.9	10089.09	0.13	0.899	3.1844	2.7261∼3.6428
Work value	9	99.7	2451.81	–1.04	0.3319	3.0836	2.8151∼3.3521
Work stress	9	99.6	2028.89	–0.61	0.5585	3.0547	2.6868∼3.4226

NO., number of included samples; E.P, P-value of Egger’s test; t, t-value reported by the Egger’s test.

## Discussion

For the first time, the research on the job satisfaction status and main influencing factors of “village doctors” in global publications was comprehensively summarized and analyzed in this systematic review. For health policy makers, the evaluation results of job satisfaction and its influencing factors are also an essential reference basis, which not only reflects the implementation effect of the current health manpower policy but is also one of the important “wind vanes” for adjusting the policy (12, 24, 61, 62). Considering China as an example, as the “gatekeeper” of the health status of more than 500 million rural residents, village doctors (63) had paid 7.9, 8.0, and 7.6 diagnosis and treatment times per day from 2018 to 2020, which was much higher than the overall status of this index of Chinese medical personnel in corresponding years (64–66). Therefore, under the huge workload, the level of job satisfaction of this group would inevitably affect its service quality and then affect the stability of the whole rural healthcare system.

A comprehensive survey of medical workers’ job satisfaction was an important factor in evaluating health policies and predicting the medical staff’s turnover rate. However, so far, few job satisfaction scales have been designed for village doctors, and most surveys were mainly applied to medical staffs in large general hospitals or urban medical institutions (16–18, 67–71). The current evidence of job satisfaction measurement for village doctors is scattered, and it is urgent to comprehensively evaluate the overall satisfaction of this group. Therefore, taking the “5-point Likert scale” as the bridge, we evaluated the overall job satisfaction level after formula conversion, which not only directly integrated and evaluated the job satisfaction of Chinese grass-roots health workers but also provided an important reference for the job satisfaction evaluation methods of medical staff in other countries or regions worldwide.

From the evaluations of 23,595 village doctors covering almost all provinces and regions in China, our results showed that the overall satisfaction level of this group was “moderate,” and it was directly reduced to the level of “dissatisfied” (2.76) after adjusting for the publication bias. The combined results of the other 4 studies (45, 48, 51, 56) that could not be converted into scores showed that less than half of the village doctors were satisfied with their current work, which further demonstrated the conclusion that the job satisfaction of this group was at the “middle and lower” level. This was in sharp contrast to the job satisfaction of doctors in secondary and tertiary public hospitals in cities or counties (72–74). A survey on the job satisfaction of 5,677 doctors in public hospitals showed that people who were “satisfied” or above accounted for 78.20% (74) of the total sample, while another study showed that the proportion of dissatisfaction in this group accounts for only 6.60% (73). In addition, a survey on job satisfaction of 638 public hospital doctors using the 5-point Likert scale also showed that the total score reached 4.02 ± 0.99, and the average score of each dimension exceeded 3.0 (72). Nonetheless, some studies have shown that the satisfaction of doctors in secondary and tertiary hospitals is relatively low (75), and this phenomenon is also found in 80% of primary healthcare workers in the United States (76).

Subsequently, for the analysis of various dimensions of job satisfaction, it seemed that financial rewards and job security were the most influential factors on job satisfaction, which was consistent with several previous findings (19, 25, 29, 46, 48). As village doctors were an important provider of rural primary medical and health services in China, the imperfection of the income and reward mechanisms of this group was greatly restricting the construction and development of rural primary human resources and forming a vicious circle, which would eventually seriously affect the service capacity of primary health service teams and make the quality of primary medical services unable to be guaranteed (3, 5, 21, 29, 46, 50). Similarly, other countries’ research also showed that low wages were the most common cause of job dissatisfaction among primary healthcare workers (PHCWs) in Ghana, for instance, which led to generate the poorly motivated staff and result poor quality services (77). Another study which explored the status of job satisfaction among rural PHCWs in Turkey has indicated that they are generally dissatisfied with their working conditions and that the overall satisfaction was moderate (3.21 ± 0.67 out of 5), while the most important predictor for it was found to be “Liking the workplace” (78). The findings about the determinants of health worker motivation in Ethiopia also supported the premise that financial factors are important ones, such as satisfaction with financial rewards (79). Compared with those with poor job satisfaction status in developing countries, other high-income developed countries showed a reversed trend. A survey of 1,174 primary care doctors aged 50 and under in the United Kingdom by Hann et al. found that most of this group expressed high job satisfaction toward their current jobs and only 11.8% of them had the turnover intention (11). Simultaneously, in a survey of 23,159 nurses in 385 hospitals in 10 European countries, Heinen et al. found that the proportion of them with significant job dissatisfaction was only 9%, and the figure was between 5 and 17% in different countries and specifically, most of them expressed the higher satisfaction toward financial rewards and job security compared to village doctors in developing countries (80). More specifically, a survey showed that during the epidemic peak of COVID-19, 20–30% healthcare staff reached the cutoff levels of disorders in anxiety, depression, and distress (81). This was bound to lead to a decline in job satisfaction. However, there was still hardly any survey on job satisfaction of village doctors during the COVID-19 pandemic.

Job security, which was closely related to economic returns, was also one of the important factors why village doctors were dissatisfied with their work, mainly including pension issues and whether there was insurance. Compared with doctors in tertiary hospitals, the most fundamental solution to the job security of village doctors was the inclusion of the authorized strength. Over the years, with the promotion of the county medical community and rural integration, village clinics have gradually changed from individual to public welfare. Village doctors had been included in the management of health centers, signed labor contracts with health centers, and changed their identities into temporary employees of village clinics. In China, most of the salaries and activity funds of village doctors with staffing came from the government, so their income was relatively stable, while contract doctors were more likely to face the possibility of non-renewal or termination (2, 5, 82, 83). At the same time, doctors with formal staffing also had a strong sense of belonging, so their turnover intention would be greatly reduced (84). Therefore, we suggest that village doctors should be included in the staffing of local public institutions.

Consequently, at the first step, we suggested that the government should increase financial subsidies for village doctors to ensure that their income level was equal to the average income level of local village cadres, teachers, and other occupations. Second, by standardizing the management of village clinics and the performance evaluation policy of health centers and village clinics, it may help to ensure the work environment of village doctors and then promote their work enthusiasm. Third, it cannot be overemphasized that carrying out vocational education and quality training for rural doctors can help to improve the overall quality level of village doctors. Finally, the government should speed up the improvement of the old-age insurance policy for this group, not only targeting to reflect their contribution to the development of health undertakings but also acknowledging the importance of their missions in the rural health service system.

Several limitations of this study should be mentioned. First, the data collection was only limited to Chinese village doctors, which may lead to region bias to some extent. However, village doctors in China have a cultural background and a robust representativeness of this group in most developing countries, and this targeted group has been widely studied in several previous publications related to this topic. In addition, due to methodological deficiency, the diversity of original survey tools in the included literature can inevitably lead to heterogeneity in this study. In view of this fact, we introduced a novel strategy to integrate all the studies reporting the current situation of job satisfaction for village doctors into consistent outcome indicators, which may help scholars to set up a unified job satisfaction evaluation scale of village doctors in the future. Finally, there was limited research on the interaction between demographics and other factors. Further studies are needed to better express how two or more determinants work together to affect the job satisfaction of village doctors.

## Conclusion

Combined with systematic review and meta-analysis, this study integrated the evidence-based health decision-making information to evaluate village doctors’ job satisfaction status. Their job satisfaction level was in the “lower middle” level, that is, in the state of “dissatisfied” or “neither satisfied nor dissatisfied.” In the issue of building a rural primary healthcare system, we selected China as a typical representative of developing countries, revealing that there is an urgent need to improve the working status of health workers in rural and remote areas, especially in the middle- and low-income countries. It could be concluded that the lack of financial rewards and job security was the most important reason for low job satisfaction. The government should increase financial subsidies for village doctors, take responsibility for their establishment, and introduce various policies to improve work security. Simultaneously, it is worthy of more explorations on the job satisfaction of village doctors under the background of COVID-19 in the future.

## Data availability statement

The original contributions presented in this study are included in the article/Supplementary material, further inquiries can be directed to the corresponding author.

## Author contributions

YC and YY: conceptualization, data analysis, writing—original draft preparation, and methodology. YW and YDW: material search. YW, YDW, and YC: data extraction. YC, YY, YW, YDW, and TD: writing—review and editing. TD: supervision, project administration, and funding acquisition. All authors have read and agreed to the published version of the manuscript.
