# Supplementary material for: Systematic and meta-based evaluation on job satisfaction of village doctors: An urgent need for solution issue
Source: Front Med (Lausanne). 2022 Aug 18;9:856379. doi: 10.3389/fmed.2022.856379 (PMC9433829; doi:10.3389/fmed.2022.856379)
Supplement: Supplementary file 1 [file Data_Sheet_1.zip › Appendix/Appendix A-Literature search strategies/Literature search strategies.docx]

**Appendix A. Literature search strategies**

**1. Database:** **China national knowledge infrastructure(CNKI)<2011 January 01 to 2021 December 01>**

[**https://kns.cnki.net/kns8/AdvSearch?dbprefix=SCDB&&crossDbcodes=CJFQ%2CCDMD%2CCIPD%2CCCND%2CCISD%2CSNAD%2CBDZK%2CCCJD%2CCCVD%2CCJFN**](https://kns.cnki.net/kns8/AdvSearch?dbprefix=SCDB&&crossDbcodes=CJFQ%2CCDMD%2CCIPD%2CCCND%2CCISD%2CSNAD%2CBDZK%2CCCJD%2CCCVD%2CCJFN)

Search Strategy:

--------------------------------------------------------------------------------

(SU%='乡村'+'农村'+'基层'+'县乡') AND (SU%='医生'+'医师'+'医务人员'+'赤脚医生') AND (SU%='工作满意度'+'收入满意度'+'薪酬满意度'+'晋升满意度'+'领导支持满意度'+'组织管理满意度') (219)

**2. Database: WANFANG<2011 to 2021>**

[**https://s.wanfangdata.com.cn/advanced-search/paper**](https://s.wanfangdata.com.cn/advanced-search/paper)

Search Strategy:

--------------------------------------------------------------------------------

(主题:"乡村" or "农村" or "基层" or "县乡") and (主题:"医生" or "医师" or "医务人员" or "赤脚医生") and (主题:"工作满意度" or "收入满意度" or "薪酬满意度" or "晋升满意度" or "领导支持满意度" or "组织管理满意度") (293)

**3. Database: China Science and Technology Journal Database (VIP)<2011 to 2021>**

[**http://qikan.cqvip.com/Qikan/Search/Advance?from=index**](http://qikan.cqvip.com/Qikan/Search/Advance?from=index)

Search Strategy:

--------------------------------------------------------------------------------

(M=(乡村 OR 农村 OR 基层 OR 县乡) OR R=(乡村 OR 农村 OR 基层 OR 县乡)) AND (M=(医生 OR 医师 OR 医务人员 OR 赤脚医生) OR R=(医生 OR 医师 OR 医务人员 OR 赤脚医生)) AND (M=(工作满意度 OR 收入满意度OR 薪酬满意度 OR 晋升满意度 OR 领导支持满意度 OR 组织管理满意度) OR R=(工作满意度 OR 收入满意度 OR 薪酬满意度 OR 晋升满意度 OR 领导支持满意度 OR 组织管理满意度)) (334)

**4. Database:** **Chinese BioMedical Literature Database(CBM)<2011 to 2021>**

<http://www.sinomed.ac.cn/zh/advancedSearch.jsp>

Search Strategy:

--------------------------------------------------------------------------------

(乡村 OR 农村 OR 基层 OR 县乡) AND (医生 OR 医师 OR 医务人员 OR 赤脚医生) AND (工作满意度 OR 收入满意度OR 薪酬满意度 OR 晋升满意度 OR 领导支持满意度 OR 组织管理满意度) (213)

**5.Database: Pubmed**

<https://pubmed.ncbi.nlm.nih.gov/advanced/>

Search Strategy:

--------------------------------------------------------------------------------

#1 Chinese[Title/Abstract] (244,558)

#2 (China[MeSH Terms]) OR China[Title/Abstract] (330,674)

#3 #1 OR #2 (479,115)

#4 (Health worker[MeSH Terms]) OR Health worker[Title/Abstract] (372,193)

#5 Health officer[Title/Abstract] (525)

#6 (Doctor[MeSH Terms]) OR Doctor[Title/Abstract] (212,813)

#7 (Physician[MeSH Terms]) OR Physician[Title/Abstract] (335,222)

#8 (Medical Personnel[MeSH Terms]) OR Medical Personnel[Title/Abstract] (571,683)

#9 (Medical worker[MeSH Terms]) OR Medical worker[Title/Abstract] (241,717)

#10 (Medical staff[MeSH Terms]) OR Medical staff[Title/Abstract] (39,708)

#11 #4 OR #5 OR #6 OR #7 OR #8 OR #9 OR #10 (845,341)

#12 Rural[Title/Abstract] (156,782)

#13 Countryside[Title/Abstract] (1,571)

#14 District[Title/Abstract] (74,278)

#15 Basic[Title/Abstract] (373,369)

#16 Fundamental[Title/Abstract] (205,116)

#17 Primary[Title/Abstract] (1,692,589)

#18 Grass roots[Title/Abstract] (776)

#19 #12 OR #13 OR #14 OR #15 OR #16 OR #17 OR #18 (2,415,719)

#20 ("job satisfaction"[MeSH Terms] OR "job satisfaction"[All Fields] OR "work satisfaction"[All Fields] OR "career satisfaction"[All Fields]) (31,080)

#21 ("0000"[Date - Publication] : "2021/12/01"[Date - Publication]) (33,393,949)

#22 #3 AND #11 AND #19 AND #20 AND #21 (85)

**6. Database: Embase<2011 to 2021>**

[**https://www.embase.com/#advancedSearch**](https://www.embase.com/#advancedSearch)

Search Strategy:

--------------------------------------------------------------------------------

(rural OR countryside OR village OR basic OR primary OR 'grass roots' OR district OR fundamental) AND (doctor OR physician OR practitioner OR 'health worker' OR 'health officer' OR 'medical personnel' OR 'health personnel' OR 'medical worker' OR 'medical staff') AND 'china'/exp AND ('job satisfaction'/exp OR 'work satisfaction'/exp OR 'career satisfaction'/exp) AND 'cross-sectional study'/exp AND [<2011-2021]/py (69)

**7. Web of Science<2011 January 01 to 2021 December 01>**

<https://www.webofscience.com/wos/alldb/summary/17fa921c-3db7-4999-85c7-993b5de0c5dc-1a3053f6/relevance/1>

Search Strategy:

--------------------------------------------------------------------------------

(TS=(rural OR countryside OR district OR village OR grassroots)) AND (TS=(doctor OR physician OR practitioner OR “health worker” OR “health officer” OR “health personnel” OR “medical personnel” OR “medical worker” OR “medical staff” OR “physician assistant”)) AND (TS=(“job satisfaction” OR "work satisfaction" OR "career satisfaction")) AND (TS=(China OR Chinese)) (34)
