# Supplementary material for: Systematic and meta-based evaluation on job satisfaction of village doctors: An urgent need for solution issue
Source: Front Med (Lausanne). 2022 Aug 18;9:856379. doi: 10.3389/fmed.2022.856379 (PMC9433829; doi:10.3389/fmed.2022.856379)
Supplement: Supplementary file 1 [file Data_Sheet_1.zip › Appendix/Appendix B-Job satisfaction evaluation results and conversion methods of each original study.docx]

| **Job satisfaction evaluation results and conversion methods of each original study** | | | | | | |
| --- | --- | --- | --- | --- | --- | --- |
| **Study ID** | **Sample Size**  **(qualified rate%)** | **Measurement of scales** | **Evaluation method of satisfaction** | **MJS** | **SJS** | |
| 1 | 2,693(96.6) | Likert 6 point | B^*^ | 3.38 | 0.93 | |
| 2 | 935(95.02%) | Likert 5 point | B | 2.28 | 0.79 | |
| 3 | 1,197(79.8%) | Likert 5 point | A^*^ | 3.64 | 0.72 | |
| 4 | 370 | Likert 5 point | B | 3.03^a^ | 0.58^a^ | |
| 5 | 495(91.6%) | Likert 5 point | D^*^ | 3.41 | 0.68 | |
| 6 | 1,221(91.19%) | Likert 4 point | C^*^ | 594 (48.6%) | | Unavailable |
| 7 | 152^**^ | Likert 5 point | D | 3.36 | 0.86 | |
| 8 | 1,472 | Likert 5 point | D | 3.19^b^ | 0.97^b^ | |
| 9 | 1,147^c^ | Likert 5 point | D | 2.87^c^ | 1.04^c^ | |
| 10 | 115^**^ | Likert 5 point | D | 3.06 | 0.85 | |
| 11 | 145(100%) | Likert 5 point | A^d^ | *NA* | *NA* | |
| 12 | 77(100%) | Likert 5 point | A^e^ | *NA* | *NA* | |
| 13 | 107(98.16%) | Likert 4 point^f^ | A | 3.26 | 0.28 | |
| 14 | 41^**^ | Likert 5 point | D | 3.93 | 0.79 | |
| 15 | 1,170(97.5%) | Likert 5 point | C | 360(30.8%) | Unavailable | |
| 16 | 120(100%) | 3-point scale | E^*^ | 2.7 | 0.41 | |
| 17 | 184(92.0%) | Likert 5 point | A^e^ | *NA* | *NA* | |
| 18 | 1,018(100%) | Likert 5 point | D | *NA* | *NA* | |
| 19 | 195(92.86%) | 2-point scale | C | 112(57.4%) | Unavailable | |
| 20 | 621(100%) | Likert 5 point | D | 2.48 | 0.86 | |
| 21 | 300(97.0%) | Likert 5 point | A^e^ | *NA* | *NA* | |
| 22 | 2,693(96.0%) | Likert 6 point | B | 3.15 | 1.25 | |
| 23 | 68(100%) | Likert 4 point^f^ | A | 4.36 | 0.26 | |
| 24 | 246(98%) | Likert 5 point | B^a^ | 3.27 | 0.36 | |
| 25 | 149(99.33%) | Likert 5 point | D | 3.44 | 0.62 | |
| 26 | 535(100%) | 3-point scale | E | *NA* | *NA* | |
| 27 | 470(94.0%) | Likert 5 point | B | 4.47 | 0.63 | |
| 28 | 100(100%) | Likert 5 point | A | 3.18 | 0.2 | |
| 29 | 1,032(95.1%) | Likert 5 point | A | *NA* | *NA* | |
| 30 | 1,018(98.36%) | Likert 5 point | D^g^ | 378(37.1%) | Unavailable | |
| 31 | 954(100%) | 3-point scale | E | 2.55 | 0.23 | |
| 32 | 335(91.0%) | 3-point scale | E | 2.75 | 0.58 | |
| 33 | 552(90.0%) | Likert 5 point | B | 3.38 | 0.43 | |
| 34 | 176^**^ | Likert 5 point | D | 3.26 | 0.56 | |
| 35 | 379(95.95%) | Likert 5 point | B | 3.03 | 0.63 | |
| 36 | 85(100%) | Likert 4 point^f^ | A | 2.25 | 0.22 | |
| 37 | 1,037(98.57%) | Likert 5 point | A | *NA* | *NA* | |

A: Satisfaction = VD% × 1.00 + DS% × 2.00 + NS% × 3.00 + ST% × 4.00 + VS% × 5.00; B: Satisfaction = reported score/maximum total score × 5.00; C: The proportional distribution were reported in not convertible scores; D: No conversion was required; E: Satisfaction = DS% × 1.50 + NS% × 3.00 + ST% × 4.50; MJS: Mean scores of overall job satisfaction; SJS: Standard deviation of overall job satisfaction; NA: Not Reported.
