# Supplementary figures and images for: Systematic and meta-based evaluation on job satisfaction of village doctors: An urgent need for solution issue
Source: Front Med (Lausanne). 2022 Aug 18;9:856379. doi: 10.3389/fmed.2022.856379 (PMC9433829; doi:10.3389/fmed.2022.856379)

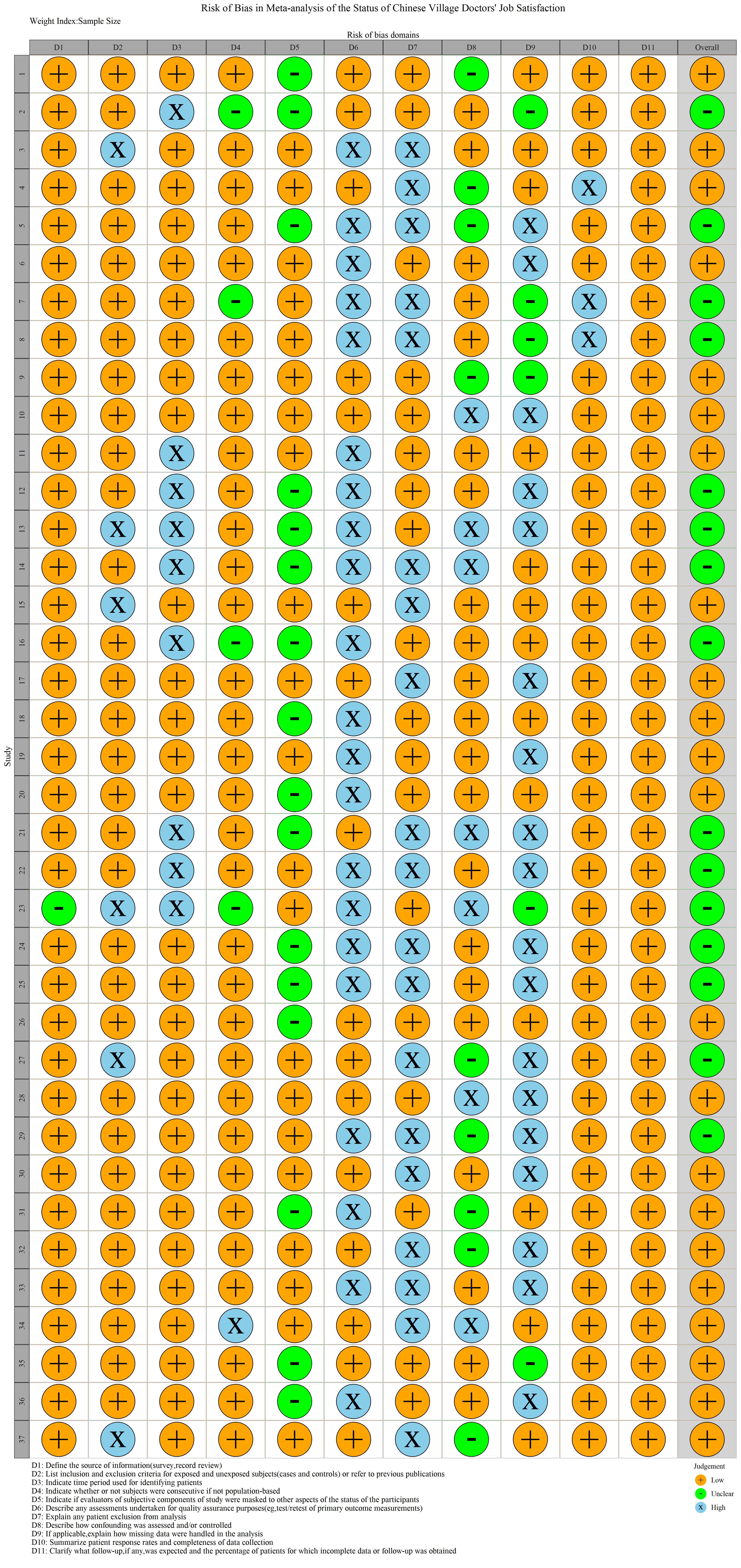

Supplement: Supplementary file 1 [file Data_Sheet_1.zip › Appendix/Appendix C-traffic light plot.png]
